# Plant Pathogenic and Endophytic *Colletotrichum fructicola*

**DOI:** 10.3390/microorganisms13071465

**Published:** 2025-06-24

**Authors:** Latiffah Zakaria

**Affiliations:** School of Biological Sciences, Universiti Sains Malaysia, USM, Gelugor 11800, Malaysia; lfah@usm.my

**Keywords:** *Colletotrichum fructicola*, endophyte, pathogen, fruit crops, ornamental, medicinal plants, tree nuts, peanuts, weeds

## Abstract

*Colletotrichum fructicola* is a member of the gloeosporioides complex and can act as a pathogen, causing anthracnose in various plants and as an endophyte residing in healthy plants. As a plant pathogen, *C. fructicola* has been frequently reported to cause anthracnose in chili fruit and tea plants, bitter rot in apples and pears, crown rot in strawberries, and Glomerella leaf spot in apples, which are the most common diseases associated with this pathogen. Over the years, *C. fructicola* has been reported to infect a wide range of plants in tropical, subtropical, and temperate regions, including various types of fruit crops, ornamental and medicinal plants, tree nuts, peanuts, and weeds. Several reports have also been made regarding endophytic *C. fructicola* recovered from different plant parts. Endophytic *C. fructicola* has the ability to switch to a pathogenic state, which may contribute to the infection of host and other susceptible plants. Due to the economic importance of *C. fructicola* infections, the present review highlighted *C. fructicola* as a plant pathogen and endophyte, providing a summary of its infections in various plants and endophytic ability to inhabit plant tissues. Several control measures for managing *C. fructicola* infections have also been provided.

## 1. Introduction

*Colletotrichum* species are mainly regarded as pathogens but have also been identified as endophytes in various plants and as saprophytes [1]. Plant pathogenic *Colletotrichum* have a wide host range, and almost all crops are susceptible to one or more species. The disease caused by *Colletotrichum* spp. is known as anthracnose, and its symptoms include leaf spot, stem rot, postharvest rot, and cankers [2,3]. Owing to its economic importance, the genus *Colletotrichum* is listed among the top 10 plant pathogenic fungi [2].

Endophytic *Colletotrichum* resides in healthy tissues of a wide range of host plants. Endophytes have been reported to provide advantages to host plants, such as promoting growth, resistance against diseases, and drought tolerance [4,5]. In certain conditions, such as during stress and senescence, endophytes can transform into pathogens [6].

*Colletotrichum* species are grouped into 15 species complexes or phylogenetic clades, namely, acutatum, agaves, boninense, caudatum, dematium, destructivum, dracaenophilum, gigasporum, gloeosporioides, graminicola, magnum, orbiculare, orchidearum, spaethianum, and truncatum [7,8,9]. A species complex is described as a group of species that form a monophyletic clade and display similar morphological characteristics [1]. Thus, to resolve the different *Colletotrichum* species complexes, as well as species within a complex, multiple loci are used as markers. Common markers used in the phylogenetic analysis of *Colletotrichum* species are actin (ACT), calmodulin (CAL), chitin synthase, glyceraldehyde-3-phosphate dehydrogenase (GAPDH), internal transcribed spacer (ITS), glutamine synthetase (GS), manganese superoxide dismutase, β-tubulin, and the DNA lyase and the mating-type locus *MAT*1-2-1 (APN2/MAT-IGS) [6,7,10,11,12,13].

*Colletotrichum fructicola* is member of the gloeosporioides complex that comprises 22 species and one subspecies [7]. Species in the gloeosporioides complex include many pathogens that cause anthracnose in various plants. Some species, including *C. fructicola*, can also act as endophytes that reside in many healthy plants. As the morphological and physiological characteristics of *C. fructicola* and other species of the gloeosporioides complex are similar, multiple markers have been used to reliably identify and distinguish them [7].

The following markers are suggested for successful species identification and phylogenetic analysis of species within the gloeosporioides complex: TUB2, GS, GAPDH, APN2, and ApMat [7,14,15] According to Vieira et al. [16] and dos Santos Vieira [17], ACT, CHS-1, ITS, and GAPDH are markers that are not suitable for species delimitation. The most suitable markers to utilize for species identification, however, are up for debate. To accurately identify species within the gloeosporioides complex, multiple markers are still needed.

In Northern Thailand, *C. fructicola* was first isolated from diseased coffee berries (*Coffea arabica*) and described by Pihastuti et al. [18]. Later, the species was isolated as a leaf endophyte of cacao (*Theobroma cacao*) in Central America [19]; however, the species was referred to as *C. ignotum*. During a reassessment of *C. gloeosporioides* taxonomy from various hosts worldwide, *C. ignotum* was synonymized with *C. fructicola* [7]. In the reassessment, both the morphological and cultural characteristics of *C. ignotum* and *C. fructicola* were similar. Based on the phylogenetic analysis of multiple loci, both species were monophyletic within the Musae clade. The type specimens of both species were also grouped into the same haplotype subgroup [7]. In addition, *Glomerella cingulata* var. *minor*, which was described from *Ficus*, was also synonymized with *C. fructicola* [7]. The ex-holotype culture of *G. cingulata* var. *minor* was found to phylogenetically match the type specimen of *C. fructicola* [7].

As a plant pathogen, *C. fructicola* has been frequently reported to cause anthracnose in chili fruit and tea plants, bitter rot in apples and pears, crown rot in strawberries, and Glomerella leaf spot in apples, which are the most common diseases associated with this pathogen. Over the years, *C. fructicola* has been reported to infect a wide range of plants in tropical, subtropical, and temperate regions, including various types of fruit crops, ornamental and medicinal plants, tree nuts, peanuts, and weeds. Several reports have also been made regarding endophytic *C. fructicola* recovered from different plant parts, which demonstrated its wide host range and worldwide distribution. The present review discusses *C. fructicola* as both a plant pathogen and endophyte, providing a comprehensive summary of the infections it causes in various plants, as well its endophytic host plants.

## 2. Plant Pathogenic *Colletotrichum fructicola*

Infections by *C. fructicola* primarily cause anthracnose in various plants. Symptoms of anthracnose commonly appear as dark lesions on infected plant parts. In severe infections, sunken lesions become visible, and during moist or wet conditions, conidial masses form in lesions. Depending on the plant part affected, anthracnose is also referred to as fruit rot, leaf blight, stem rot, crown rot, seedling blight, and twig blight [20,21]. Typically, *C. fructicola* grows as a saprophyte in soil and plant debris and infects plants through the water-splashing of conidia and air dissemination of ascospores [22].

Since the first description was made for *C. fructicola* isolated from coffee berries [18], this species has been identified as a pathogen in various other plants worldwide. Plant pathogenic *C. fructicola* not only causes anthracnose but also other diseases, such as bitter rot, crown rot, leaf spot, and leaf blotch. After the taxonomy of species within the gloeosporioides complex was revised, the *C. fructicola* reported as a pathogen was identified using multiple markers and morphological characteristics [7,9,18,21]. The pathogenicity of *C. fructicola* isolates was also documented, indicating the impact they have on production (Tables 1–15).

The species-specific detection of plant pathogenic *C. fructicola* using several PCR-based methods has been developed to facilitate detection and differentiation from other *Colletotrichum* species. A single marker derived from comparative genomics analysis was developed to differentiate *C. fructicola* among prevalent pathogens of strawberry [23]. A quantitative PCR assay employing ITS primers was utilized to detect *C. fructicola* in the leaves of tea oil trees [24]. Specific primers and hydrolysis probes were developed as components of a real-time PCR assay aimed at detecting *C. fructicola* associated with bitter rot in apples [25]. These PCR-based methods offer considerable benefits for the swift detection of *C. fructicola*, contributing to improved disease management.

Many types of plants are infected by *C. fructicola*, including agricultural crops, ornamental and medicinal plants, cacti, and weeds. The host range has broadened over time. This was likely due to species identification using molecular markers, of which several species of *C. gloeosporioides* sensu lato were phylogenetically described since the taxonomic revision of the gloeosporioides complex. Other possibilities may be related to its pathogenic and endophytic lifestyles. Moreover, the availability of host plants and suitable climatic conditions are favorable for *C. fructicola* growth and development, particularly in tropical and subtropical regions.

Several reasons may be attributed to the introduction and dispersal of *C. fructicola* into new areas, such as via the agricultural and plant trade of latent pathogens or endophytes, transportation, and through human-aided means [26,27]. These methods of the introduction and dispersal of fungal pathogens are also known as hitchhiking, in which hitchhikers are commonly hidden in a plant in the form of small fungal propagules [28,29]. Hitchhiking of fungal pathogens was reported to explain the introduction of *Diaporthe* spp. into new areas through the international trade of plant materials [30]. Fungal propagules can infect and establish host plants in the new environment due to host availability and climate suitability. The establishment of *C. fructicola* in some parts of European Union countries is associated with these two factors [31].

The ability of latent or endophytic *C. fructicola* to hitchhike poses epidemiological concern due to their hidden nature, which facilitate their undetected dissemination and subsequent emergence, potentially resulting in disease outbreak in a new environment. Once introduced, latent or endophytic *C. fructicola* can occasionally switch hosts, transitioning from their original host plants to either closely related or even more distantly related plants [32]. Furthermore, lack of host specificity of *C. fructicola* allows them to infect a variety of crops.

## 3. Common Fruit Crops Infected by *Colletotrichum fructicola*

Fruit crops are susceptible to *C. fructicola* infections, which result in anthracnose, fruit rot, crown rot, and leaf spot. Some of these fruit crops, such as apples, pears, citrus, strawberries, avocados, mangoes, papaya, dragon fruits, and pineapples, are economically important in several crop-producing countries. The pathogen not only infects fruits but also other plant parts, including the leaves, petals, petioles, and stems. The most noticeable anthracnose symptoms appear on the surface of fruits, manifesting as brown-to-black sunken lesions containing masses of conidia [33]. Dark, sunken lesions may also appear on the leaves and stems. Table 1 summarizes common symptoms caused by *Colletotrichum* on fruits, leaves, and stems.

Glomerella leaf spot on apples (*Malus domestica*) is a serious disease caused by *C. fructicola*, which often occurs in apple-producing countries (Table 2), in subtropical and humid environments, but is confined to a number of countries in Asia and America [34,37,38,39]. Bitter rot in apples is often associated with *C. fructicola*, and the disease occurs worldwide (Table 2). Apple bitter rot affects all cultivars, although they have different susceptibilities [40]. The pathogen infects fruits in orchards and after harvest during storage [41]. In addition to Glomerella leaf spot and bitter rot, *C. fructicola* causes apple anthracnose [42,43,44,45] and fruit rot [46].

Anthracnose and ripe rot can occur in *C. fructicola*-infected grapes (*Vitis vinifera* and other *Vitis* spp.) in several grape-producing countries, including China, Brazil, Korea, and Japan (Table 3). Initially, anthracnose in grapes was caused by *Elsinoe ampelina*, but later *Colletotrichum* spp., including *C. fructicola*, were found to be associated with the disease [54,55]. The anthracnose symptoms exhibited by *E. ampelina* and *Colletotrichum* spp. are similar, resulting in sunken, necrotic lesions on the berries, leaves, shoots, and stems [56]. The most prevalent species recovered from grape anthracnose in Zhejiang Province, China, was *C. fructicola* [57]. In addition to anthracnose, *C. fructicola* also causes ripe rot in grape berries [58]. In Southern Brazil, *C. fructicola* was the second-most dominant species causing ripe rot in grapes [59].

After apples and grapes, pears (*Pyrus* spp.) are important temperate fruit crops broadly cultivated in China, Argentina, USA, Spain, and Italy. In a study by Fu et al. [64], *C. fructicola* was the most common species associated with infections of the fruits and leaves of *P. pyrifolia* and *P. bretschneideri* in six main pear cultivation provinces in China. Other reports of *C. fructicola* as a pathogen of pears are presented in Table 4. In pear fruits, *C. fructicola* can cause bitter rot and anthracnose. Anthracnose caused by *C. fructicola* in hybrid pear fruits (*P. pyrifolia* × *P. communis*) was reported in Korea. Black specks lesion on the fruits that become larger lead to fruit drop. On pear leaves, three types of symptoms appear to be associated with *C. fructicola* infections, namely large, necrotic lesions, small round spots, and tiny black spots [64,65,66].

In *Citrus* spp., *C. fructicola* can cause anthracnose in the fruits, leaves, stems, and petals (Table 5). Various *Citrus* spp. have been previously reported to be infected by *C. fructicola*, including *Ci. bergamia* (bergamot orange), *Ci. grandis* (pomelo), *Ci. reticulata* (mandarin orange), and *Ci. sinensis* (oranges), as well as kumquat (*Fortunella margarita*), during the preharvest and postharvest periods [69,70,71,72].

Strawberries (*Fragaria* × *ananassa*) are susceptible to anthracnose infection, which commonly occurs soon after planting and at the seedling stage [76]. The most prevalent species isolated from the infected plant parts of strawberries [77,78] including the fruits, leaves, petioles, and stolons, was reported to be *C. fructicola* (Table 6). Based on a study by Gan et al. [79], *C. fructicola* was the main virulent species found in the field, although less-virulent isolates were also detected. Strawberry crown rot is also caused by *C. fructicola* (Table 6), resulting in the wilting and stunting of aboveground plant parts, which can cause the plant to collapse. Cutting into the crown tissues revealed reddish-brown lesions [80,81,82].

Notable anthracnose infections of fruit crops caused by *C. fructicola* include a number of tropical and other fruit crops, such as kiwis, cherries, litchi, carambola, and pomegranates (Table 7). Shot hole leaf caused by *C. fructicola* was reported in *Prunus sibirica* [89].

### Other Host Plants Infected by Colletotrichum fructicola

Various other plants, including coffee berries, *Camellia* spp., vegetable crops, ornamentals, peanuts, tree nuts, medicinal plants, and weeds, can also be infected by *C. fructicola*.

Anthracnose in coffee berries is caused by a complex of *Colletotrichum* spp., including *C. fructicola* (Table 8). Pathogenic *C. fructicola* associated with diseased coffee berries was first reported in Northern Thailand [18]. Another report of *C. fructicola* infection associated with coffee berries was made in Puerto Rico, and the resulting disease was referred to as coffee fruit rot [120]. The disease affected the maturity stages of coffee berries and caused internal and external rotting of green berries. Severe internal rot was incited by *C. fructicola* and the berries later become mummified [120,121].

Leaf anthracnose is a serious disease that affects *Camellia* spp. [122,123,124]. Pathogenic *C. fructicola* has been found to cause leaf anthracnose in *Ca*. *sinensis*, a beverage crop, as well in the tea oil trees, *Ca*. *oleifera* and *Ca*. *yuhsienensis*, which would lead to economic losses (Table 9).

Anthracnose in several vegetable crops is often caused by *C. fructicola*. In chili, anthracnose mainly affects the fruits but can also infect leaves (Table 10). Anthracnose caused by *C. fructicola* has been detected in Japanese pickling melons, culinary melons, luffa sponge gourds, and Chinese flowering cabbages (Table 10).

The leaves of various ornamental plants can be infected by *C. fructicola*, causing leaf spot or anthracnose (Table 11). However, in *Phalaenopsis*, *C. fructicola* can infect the petals, causing a ring of green tissue that borders necrotic areas [145]. Infected leaves eventually wither and can lead to defoliation, as observed during infections of *Bletilla striata* [146] and *Salix babylonica* [147].

Peanuts and tree nuts are also susceptible to *C. fructicola* infection (Table 12). In peanuts, *C. fructicola* can cause leaf spot similar to that observed during the infection of walnuts, pecan, macadamia, and Chinese hickory. Fruit anthracnose has been reported in walnuts and pecan [160,161]. Infection on the fruits can lead to mummified fruits and fruit drops.

Various types of medicinal plants can be infected by *C. fructicola*, causing leaf spot and stem rot (Table 13). Infection causes wilting and defoliation of the leaves [165,166,167,168,169,170,171].

Weeds can also be infected by *C. fructicola*, mainly causing leaf spot (Table 14). Besides leaf spot, the pathogen can also cause stem rot, crown rot, and petiole rot in the aquatic weed, water hyacinth (*Eichhornia crassipes*) [172]. Pigweed and Japanese brome can also be infected by *C. fructicola* [173,174]. The infected parts turn yellow, thinner, and eventually wither [172,174].

Other plants and crops that can be infected by *C. fructicola* are presented in Table 15. Leaf spot or anthracnose is the most commonly reported disease for these plants/crops.

## 4. Infection and Colonization by *Colletotrichum fructicola*

A similar infection and colonization behavior is shared between *C. fructicola* and other species of the gloeosporioides complex. The penetration and colonization of *C. fructicola* have been characterized in apples (*M. domestica* Gala) and Ca. *oleifera* leaves [194,195]. Penetration of the host begins with the germination of conidia and the formation of appressoria, whereafter the penetration peg expedites the entry of the pathogen through the epidermis and cuticle of the host [196,197]. In *C. oleifera*, the pathogen can also infect or invade through the stomata via germ tubes or hyphae [195]. After penetration, infection vesicles develop within the epidermal cells, followed by the formation of primary hyphae that spread into adjacent cells.

**Figure 1:**
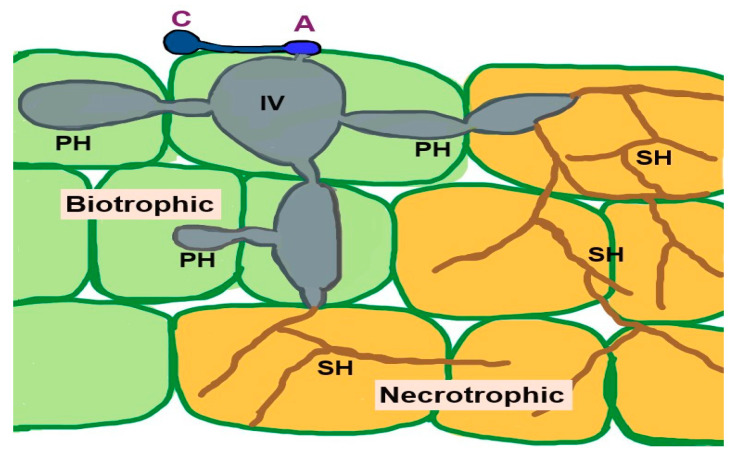
*Colletotrichum fructicola* infection on susceptible host plant. Biotrophic stage: Conidium (C) on the host plant germinate and produce an appressorium (A) that assists in the penetration of the host tissues and forms an infection vesicle (IV) and primary hypha (PH) in the living tissues. During this stage, the host cell maintains its structural integrity. The fungus then transitions to the necrotrophic stage by developing thin secondary hyphae (SH), colonizing and killing the surrounding tissues. The fungus produces cell wall degrading enzymes, proteases, and nutrient transporters to aid in tissue degradation and nutrient acquisition from the dead host cells [198].

The pathogen remains in living plant tissues and is established therein by absorbing nutrients without killing the plant cells, which demonstrates the biotrophic stage. Subsequently, secondary hyphae form, initiating the necrotrophic stage. At this stage, the host tissues are destroyed, and symptoms of disease emerge [198,199]. Figure 1 shows the infection of *C. fructicola* on susceptible host plants with biotropic and nectrotropic stages.

The latent or quiescent period is a transitional stage that occurs towards the biotrophic and necrotrophic stages. The latent period is important for species associated with postharvest diseases, particularly fruit rot or anthracnose. Pathogenic *Colletotrichum* remains dormant within the plant host before shifting to the active stages. The transition from dormant to biotrophic and necrotrophic stages occurs during fruit ripening in response to biochemical and physiological changes in the fruits [200].

*Colletotrichum* employs a latent period as a means of survival, waiting for the host to become susceptible following harvest. Since symptoms are not immediately visible, infected crops pass through sorting and inspection stages, entering the supply chain without detection [201]. The pathogen’s ability to remain latent allows it to be transported over long distances in crops that appear healthy, facilitating its dissemination to new areas. When the crops encounter stress factors, like mechanical damage, temperature and humidity changes, the pathogen switches from a latent state to active growth stages, rapidly multiplying and spreading during storage or transport, particularly in packed conditions with high moisture and limited airflow [202]. The appearance of symptoms during postharvest indicate the beginning of a postharvest disease outbreak.

**Figure 2:**
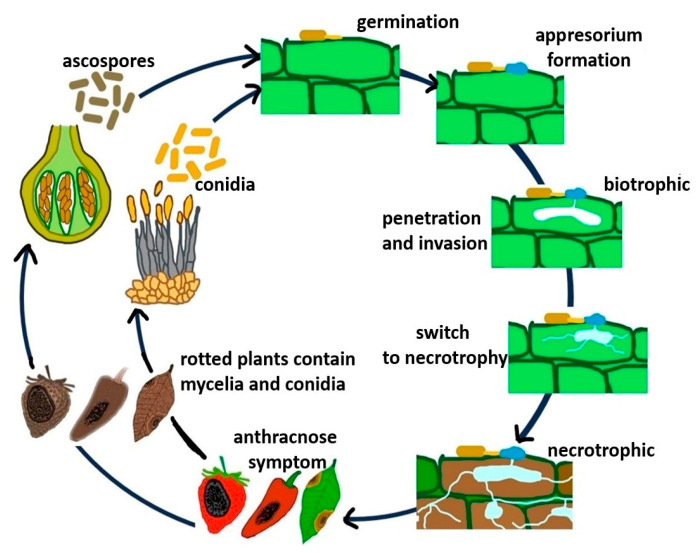
Life cycle of *C. fructicola* and disease development on infected plants.

The *Colletotrichum* life cycle includes asexual and sexual stages. During pathogen growth, acervuli develop in plant tissues and produce masses of conidia. These conidia serve as the primary inoculum during the new infection cycle and are dispersed to new hosts or the same host plant in a new location via rain splashes, irrigation water, wind-driven rain, and cultivation tools [203]. The perithecia and sexual fruiting structures are formed under favorable conditions, which release and disperse ascospores. In the absence of a susceptible host, the perithecia act as survival structures [18,19]. Figure 2 shows the life cycle and disease development of *Colletotrichum* spp., which is also applicable to *C. fructicola*. A detailed review of the *Colletotrichum* spp. life cycle was previously described by Latunde-Dada [204] and de Silva et al. [203].

## 5. Molecular Pathogenesis of *Colletotrichum fructicola*

The molecular pathogenesis of *C. fructicola* in infected host plants has been studied in several plants, including tea oil trees (Ca. *oleifera*), strawberries, apples, and pears, at different stages of infection. In these studies, structure-specific genes, virulence factors, effector proteins, secreted proteases, transcription factors, and secondary metabolite enzymes involved in host–pathogen interactions were identified, and in some cases, the genes or proteins characterized. Data on the molecular mechanisms underlying *C. fructicola* infection provide insights into disease pathogenesis, the colonization of the pathogen, and the defense mechanisms of the plant. Some studies on the molecular mechanisms of *C. fructicola* infection have been performed on *Ca. oleifera* anthracnose, Glomerella leaf spot in apples, strawberry anthracnose, and pear anthracnose.

### 5.1. Pathogenesis of Colletotrichum fructicola

Based on comparative genome analysis, the *C. fructicola* genome contains the largest number of virulence factors, including plant cell wall-degrading enzymes, secondary metabolite biosynthetic enzymes, effectors, secreted proteinases, and small secreted proteins [205]. These virulence factors are expressed at different stages of the infection process to ensure successful colonization, starting before penetration, after appressoria penetration, during colonization by intracellular hyphae, and when switching from biotrophy to necrotrophy [206]. At different stages of infection, a range of genes and proteins are involved in plant cell wall degradation and secondary metabolism, during which effector expression is highly upregulated [207]. Several genes and proteins involved in the pathogenicity of *C. fructicola* have been identified and characterized, as presented in Table 16.

The transcriptomic profiles of differentially expressed genes in the conidia, appressoria, and infectious hyphae of *C. fructicola* during infections in apple leaves were previously obtained. In *C. fructicola*-strawberry interactions, during plant cell wall degradation and penetration into plant tissues, genes encoding putative effectors, including the chitin-binding protein, were upregulated. The detection of chitin-binding protein at this stage might indicate that the pathogen attaches to the host by expressing chitinases or the chitin-binding protein [207,216]. During the early stage of strawberry infection, several pectin-degrading enzyme families were detected, including the carbohydrate esterase family 1, glycoside hydrolase family, and several pectate lyases. Cutinase-encoding genes were also upregulated [207].

For successful infection, pathogens produce numerous virulence effectors that facilitate infection and exploit plant physiology and immune responses [217,218]. Comparative genome studies have shown that *Colletotrichum* species contain hundreds of putative effectors that are unique, conserved, and vital for pathogen adaptation in the host plant [206,219,220].

Effectors are highly expressed during *C. fructicola* infection and host plant colonization. In hemibiotrophic infections caused by *Colletotrichum*, effectors are induced by the host plant and successively expressed. Most of these effectors are expressed during the early stage of host penetration by the appressorium and at the beginning of the necrotrophic stage, where cell death is induced, hastening a switch between the stages of infection [221]. During *C. fructicola* infection in apple leaves, many candidate effector proteins were detected, and their expression strongly induced. Several of these effector proteins were also upregulated during *C. fructicola* infection in strawberries [195]. A novel effector, CfEC92, was identified to be involved in *C. fructicola* infections causing leaf spot in apples and is thus considered important for pathogen virulence. CfEC92 expression was induced during the early stages of infection and the formation of appressoria to assist the pathogen in penetrating the host plant and inducing vesicle formation at the biotrophic stage [194].

The significant role of effectors in the pathogenicity of *Colletotrichum* positions them as potential targets for developing disease management strategies, such as plant breeding and biocontrol [222]. Certain effectors are conserved among various *Colletotrichum* species and play a crucial role in their overall virulence. These effectors are potential targets for resistance that might provide protection against multiple *Colletotrichum* species. For example, the conserved effector NIS1 has demonstrated its importance for virulence in multiple *Colletotrichum* species [223]. Several *Colletotrichum* effectors that exhibit antimicrobial properties against competing microbes have been discovered. Although these facilitate the colonization of the fungi, they also offer a promising source of new antimicrobial substances for biocontrol agents. For instance, the ribonucleases CfRibo1 and CfRibo2 from *C. fructicola* function as antimicrobial effectors, suggesting their potential application as antimicrobial effectors [224].

Transcription factors have been reported to play a role in the pathogenesis of *C. fructicola* causing Glomerella leaf spot in apples. During the pathogenesis of *C. fructicola* in apple leaves, the expression of the transcription factor *CfSte12* was found to be upregulated throughout the germination of conidia, formation of appressoria, penetration, and development of structures required for sexual reproduction [209]. Another transcription factor, CfMcm1, was expressed during early infection and in conidia. The transcription factor, CfMcm1, is the main regulator that plays a role in pathogenicity, sexual and asexual reproduction, and melanin synthesis [213]. Moreover, the transcription factor, CfCpmd1, is important for strain compatibility in sexual reproduction, as it affects the growth of hyphae, sporulation, and the formation of appressoria, which play a notable role in the pathogenicity of *C. fructicola* causing leaf spot in apples [215].

Secondary metabolites also contribute to *C. fructicola* pathogenicity. During the pathogenesis of *C. fructicola* in strawberries, the expression of secondary metabolite backbone genes was upregulated, of which polyketide synthase-encoding genes involved in melanin biosynthesis were detected. Melanin biosynthesis plays an essential role in the development of appressoria during pathogenesis [207]. Other secondary metabolite backbone genes were also found to be highly regulated 96 h after inoculation, which corresponds with the necrotrophic stage of the pathogen [207]. Understanding the pathogenesis and interaction between *C. fructicola* and the host plant is pivotal for strategizing suitable disease management and crop protection practices.

### 5.2. Host Defense

During infection and colonization, *C. fructicola* encounters several defense responses by the host plant. Studies on the mechanisms of defense in response to *C. fructicola* pathogenesis have been conducted for strawberries, tea oil trees, and pears, providing insight into the defense strategies employed by host plants.

The pathogenesis of *C. fructicola* in strawberry and pear leaves is generally similar, with salicylic acid, jasmonic acid, reactive oxygen species, and peroxidases playing roles in pathogen resistance. Salicylic acid is the main plant defense hormone used against biotrophic pathogens, whereas jasmonic acid and ethylene are essential for resistance against necrotrophic pathogens [225]. The genes associated with salicylic and jasmonic acid biosynthesis and signaling pathways were upregulated during infection in strawberry and pear leaves, demonstrating that both plant hormones may constitute the core defense mechanism used by host plants [207,226,227].

Peroxidases are a notable class of pathogenesis-related proteins that are activated in host plants upon pathogen infection. Seven peroxidase-encoding genes were upregulated at 96 h postinfection by *C. fructicola* in strawberries, which may indicate the activation of plant defense mechanisms [207]. Peroxidases are expressed to restrict pathogen infection by forming structural barriers or producing a toxic environment through the activation of reactive oxygen species [228].

Infection by pathogens can cause oxidative stress due to the accumulation of reactive oxygen species in plants after pathogen recognition, which represents one of the earliest activated defense responses against pathogens [229]. Reactive oxygen species were found to be regulated, and genes involved in the necrotrophic stage of strawberry infection were identified [207]. The generation of reactive oxygen species often leads to symptoms that induce cell death [207,230]. In pear leaves infected with *C. fructicola*, genes related to lignin and flavonoid biosynthesis were also found to be upregulated, demonstrating that lignin and phytoalexin are involved in the defense pathway of the host plant [227].

Flavonoids and lignin are among the products of phenylpropanoid metabolism and are involved in plant defense against pathogens [231]. Genes related to flavonoid and lignin biosynthesis were upregulated at the necrotrophic stage of *C. fructicola* infection in pear leaves. These findings indicate that lignin and phytoalexin are involved in the defense pathway utilized by pear leaves in response to pathogen infection [227].

The brassinosteroid, 24-epibrassinolide, which is a steroid-type phytohormone, may activate lignin biosynthesis in tea plants (Ca. *sinensis*) during infection by *C. fructicola* causing anthracnose. Gene analysis has shown that 24-epibrassinolide activates genes related to lignin biosynthesis in response to *C. fructicola* infection [232]. Lignin accumulation in leaves potentially strengthens the cell wall structure and reduces *C. fructicola* infections, thereby enhancing tea plant resistance [232].

Transcription factors are also involved in host plant defense against fungal pathogens. WRKY transcription factors are among the largest families of transcriptional regulators in plants that trigger plant immune responses by inactivating defense-suppressing WRKY proteins [233]. Genes encoding WRKY factors have been identified during *C. fructicola* infection in strawberries, and several genes were found to be upregulated during the mid-to-late stages of infection [207]. In a study by Li et al. [195], multiple candidate WRKYs were found to be induced by *C. fructicola* in infected tea oil trees.

The infection response showed that plants employ various mechanisms of defense against *C. fructicola*. However, the pathogen utilizes a series of antagonistic mechanisms to successfully infect and colonize host plants.

## 6. Cross-Pathogenicity

Many *Colletotrichum* species, including *C. fructicola*, can infect various plants because they are not host-specific. Studies on *C. fructicola* infecting various hosts, often as first reports, have increased over the years, showing that many host plants are susceptible to *C. fructicola* infections and may also indicate cross-infection [234,235,236,237]. Cross-infection among species within the same species complex can lead to serious infections in the plant host. Therefore, the assessment of cross-infection in various host plants could provide insights into the host range and pathogenic potential of *C. fructicola*. Table 17 summarizes reported cross-infection of *Colletotrichum* spp. from primary host to other plants.

Cross-infection of species belonging to the gloeosporioides complex has been reported in several studies [135,234,236,238]. In a study by Lakshmi et al. [238], isolates of *C. gloeosporioides* recovered from acid lime, cashew, custard apple, guava, and pomegranate were able to infect mango fruits and leaves, demonstrating cross-infection among the fruit crops. The *C. gloeosporioides* isolated from avocado fruits was pathogenic to mango fruits but showed a lower aggressiveness. Similarly, *C. gloeosporioides* isolated from mango fruits were pathogenic to avocado fruits but with a lower aggressiveness [220].

A cross-infection study on five *Colletotrichum* species, including *C. fructicola*, of several fruit crops and chili fruits, such as papaya (*Carica papaya*), orange (*Ci. reticulata*), rose apple (*Eugenia javanica*), mango (*Mangifera indica*), and guava (*Psidium guajava*), indicated that *C. fructicola* and other species of the gloeosporioides complex could infect all the fruit crops and chili fruits tested. The cross-infection study also showed that the tested species had a wide host range [236]. In a study by Freeman et al. [239], cross-infection by *C. gloeosporioides* isolated from *Limonium* occurred when tested on mangoes, strawberries, peaches, pears, and nectarines.

According to a study by Eaton et al. [240] on *Colletotrichum* spp. isolated from the anthracnose of apples, strawberries, and blueberries, mixed-fruit orchards may expedite the cross-infection of *Colletotrichum* spp. The study findings were in accordance with those of a cross-infection study by Liu et al. [135], which involved *Colletotrichum* spp., including *C. fructicola*, from chili fruits and showed that they were also able to infect pear fruits. In Sichuan Province, China, chili plants are often planted in pear orchards in which the *Colletotrichum* spp. associated with chili plants have the potential to cross-infect pear fruits [135].

The implications of cross-pathogenicity and the latent infection of *Colletotrichum* are particularly important in mixed-cropping systems. In mixed-cropping systems, certain crops may serve as asymptomatic hosts for *Colletotrichum*, harboring latent infections and elevating disease risk. These crops can serve as pathogen reservoirs, maintaining the pathogen populations and enabling carryover between seasons or plantings. On the other hand, if a crop is resistant or less susceptible, it may function as a barrier to the spread of disease [243].

Many species of the gloeosporioides complex, including *C. fructicola*, can cross-infect various hosts, as these pathogens generally have the potential to adapt to new hosts and cause infections [241,242,244,245]. However, virulence and aggressiveness may differ due to the adaptation of the pathogen in the new host and their capability to overcome host defense mechanisms [245]. Host range information from the occurrence of cross-infection provides useful data for suitable disease management methods in the field, as well as after harvest. This information will be useful for the development of resistant cultivars.

## 7. Endophytic *Colletotrichum fructicola*

The genus *Colletotrichum* comprises the most common endophytic fungal species found within plant tissues [19,246,247,248]. For instance, endophytic *Colletotrichum* spp. that are members of the gloeosporioides complex were the most common endophytes isolated from forest leaves [199,249]. Endophytic *C. fructicola* has been recovered from several plants, including citrus, grasses, mangoes, coffee, tea plants, cocoa, and aquatic plants (Table 18).

Endophytic *Colletotrichum* that inhabits host tissues may benefit the plants because endophytes can enhance plant growth, confer heat and drought tolerance, and provide disease resistance [249,250,251,252]. Based on several factors, including wounding, changes in plant physiology, host senescence, and changes in environmental factors, endophytic *Colletotrichum* may transform into a saprophyte or pathogen that induces disease symptoms [253,254,255,256,257]. According to Crouch et al. [257], endophytism of *Colletotrichum* spp. may serve as a vital part of their lifecycle; however, this is not fully understood.

**Table 18:** Endophytic *Colletotrichum fructicola* isolated from various plants.

Host Plants	Plant Parts	Countries	References
Citrus			
*Citrus reticulata* ‘Nanfengmiju’	Leaves	Jiangxi Province, China	[70]
*Fortunella margarita*	Branches	Guangxi Province, China	[70]
Grasses			
Dwarf napier (*Pennisetum purpureum*) and lemon grass (*Cymbopogon citratus*)	Leaves and sheaths	Muang District, Chiang Rai, Thailand	[258]
Lawn grass (*Axonopus compressus*)	Leaves	Penang, Malaysia	[259]
Coffee (*Coffea arabica*)	Berries	Chiang Mai, Thailand	[18]
Cacao (*Theobroma cacao*)	Leaves	Panama	[19]
Mango (*Mangifera indica*)	Young and mature leaves, stem fragments (with cork layer), and mature inflorescences	Pernambuco State, Brazil	[260]
Tea (*Camellia sinensis*)	Leaves	Fujian, Guizhou, Henan, Jiangxi, Sichuan, Yunnan, and Zhejiang, China	[6]
*Dendrobium* spp.	Leaves and roots	Mae Fah Luang District, Chiang Rai, Thailand	[261]
*Licania tomentosa*	Leaves	Brazil	[262]
Java plum (*Syzygium cumini*)	Fruits and seeds	Malang, East Java, Indonesia	[263]
*Tabernaemontana heyneana* (medicinal plant)	Leaves	Southern Western Ghats, India	[264]
*Magnolia candolli* and *M. garrettii*	Leaves	*M. candolli*: Yunnan Province, China *M. garrettii*: Chiang Mai Province, Thailand	[265]
Aquatic plants	Leaves	Yunnan, Guizhou, and Sichuan Provinces, China	[266]
Chinese boxthorn (*Lycium chinense*)	Leaves and fruits	South Chungcheong Province, Republic of Korea	[267]

Endophytic *Colletotrichum* that colonize roots, stems, and leaves, and extensively occupy plant tissues, can be categorized as Class 2 endophytes based on the description by Rodriguez et al. [256]. Class 2 endophytes are described as endophytic fungi that colonize plants through direct penetration or via infectious structures and colonize plant tissues intercellularly, with little to no effect on the host plant [256,268,269]. In healthy plants, fungal sporulation is low; however, during senescence, fungi rapidly sporulate and emerge [270].

Several studies have demonstrated that species isolated as endophytes, including *C. fructicola*, can switch to a pathogenic state [99,259,260,265,271]. These findings highlight the lifestyle-switching from endophyte to pathogen, which may contribute to the infection of various hosts by *C. fructicola*. The switching of fungal lifestyles is described as a symbiotic continuum, in which the interaction involves a balance of antagonism [272,273,274]. When endophytism appears to be in a labile state, frequent switching from endophytism to pathogenicity, or from mutualism to parasitism, occurs [275].

Pathogenic species of the gloeosporioides complex may likely switch to an endophytic state, especially the species with a wide host range. This observation was based on studies by Redman et al. [276] and Redman et al. [251]. Pathogenic *C. magna* can infect cucurbits, causing anthracnose, but can also act as an endophyte when colonizing various noncucurbits. In contrast, mutant *C. magna* were found capable of infecting tomatoes, a nonhost, and colonizing cucurbit cultivars without inducing disease symptoms, which indicated its endophytic lifestyle or mutualistic effects [260,276]. The mutation of a single locus in pathogenic *C. magna* allowed the plant pathogen to switch to an endophytic state [277]. According to Redman et al. [251], environmental factors or host plant genetic variations may play a role in causing individual isolates of pathogenic *Colletotrichum* spp. to adopt an endophytic lifestyle in host plants and provide disease resistance, drought tolerance, and growth enhancement.

**Figure 3:**
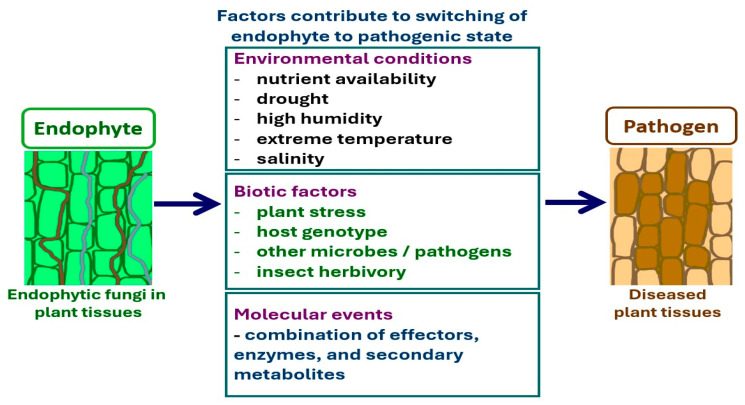
Factors contribute to lifestyle switching from endophyte to pathogen.

Although the major factors that cause lifestyle switching are difficult to determine, several have been predicted thus far, such as environmental factors and host plant genetic variations (Figure 3). Hazardous environmental conditions affect plant health, resulting in a weakening of plant defense responses. In turn, endophytes can grow abruptly and switch to a pathogenic form [278,279]. The host plant and microbial genotypes that are involved in fungal gene expression and plant-host recognition are regarded as important factors that influence whether fungi adopt a particular lifestyle [280]. In addition, higher nutrient conditions have been recognized to stimulate lifestyle switching [281]. Other factors, such as plant age and abiotic environments, have also been proposed to influence whether fungi act as pathogens or endophytes [273,274]. Under these conditions, the balance of antagonism between the endophyte and host plant is affected, resulting in disease occurrence and visible symptoms [274].

The ecological role of endophytic *C. fructicola* can be understood through a comparative study with known beneficial endophytes, such as *C. tofieldiae* [282] and *Epichloë* sp. [283]. This comparative study would include genomic and transcriptomic analyses to examine, among other factors, the presence or absence of virulence factors, genes related to stress tolerance, profiling of secondary metabolites, and pathways for phytohormone synthesis. Such analysis could uncover regulatory mechanisms that dictate whether the fungus acts as a pathogen or remains endophytic; it would also identify the genes linked to pathogenicity and those associated with beneficial traits for host plants [284].

## 8. Fine Line Between Pathogenesis and Endophytism

*Colletotrichum* spp. can exhibit both pathogenic and nonpathogenic lifestyles, demonstrating a fine line between pathogenicity and endophytism, as demonstrated in studies by Redman et al. [276] and Redman et al. [251]. The wildtype *C. magna* isolate was pathogenic to cucurbits; however, disruption of the pathogenicity gene resulted in a nonpathogenic, symbiotic *C. magna* mutant that expressed either commensal, mutualistic, or intermediate-mutualistic lifestyles [276]. Previously identified as a commensal on peppers and tomatoes in disease-resistance experiments, *C. gloeosporioides* was found to switch to mutualism in both host plants during drought and growth studies. Furthermore, *C. musae* was designated as an intermediate mutualist in disease studies on the tomato varieties, Big Beef and California Wonder, but found to be a commensal on both tomato varieties in growth enhancement experiments, as well as a mutualist on peppers in a drought-tolerance study [251].

Redman et al. [251] concluded that the host plant determines the outcome of fungal-plant symbiosis, as well as the fungal lifestyles expressed. This may be due to differences in fungal gene expression in response to the host plant or differences in the ability of a plant to respond to fungal infection. Redman et al. [251] also pointed out that slight genetic differences between plant cultivars of a species may significantly alter the outcome of fungal-plant symbiosis. Based on these findings, *Colletotrichum* infections have several possible outcomes; however, predicting lifestyle switching based on fungal-plant interactions remains difficult.

The switch from endophyte to pathogen involves significant changes in fungal gene expression, protein synthesis, and metabolic functions, which can be uncovered through comparative multi-omics approaches [285]. By identifying the genes and pathways that influence host interaction, nutrient uptake, and stress responses at various stages, multi-omics approaches facilitate the development of more focused and sustainable strategies for disease management. Multi-omic studies have been utilized to elucidate the mechanisms underlying endophytic and pathogenic states of *Diaporthe* [30] which are relevant to *C. fructicola*. Hilario and Goncalve [30] outlined the molecular mechanisms involved in the transition from endophyte to pathogen, including MAPK pathways, small RNAs, and systems for effector secretion.

Both transcriptomic and genomic approaches can yield valuable insights into the fungal switching from an endophytic to a pathogenic state. The authors of [206] conducted a comparative genomics and transcriptomics study that revealed that effectors and enzymes related to secondary metabolism are activated during the endophytic state, while hydrolases and transporters are upregulated during the transition to necrotrophy or the pathogenic state. Given the dual lifestyle of *C. fructicola,* omics technologies could be employed in the future to uncover candidate genes essential for sustaining endophytic relationships with plants and to elucidate the mechanisms that drive transitions to pathogenicity.

### Relatedness Between Endophytic and Pathogenic Lifestyles

A phylogenetic approach was used to analyze the general relationship between fungal lifestyles. From the phylogenetic analysis, fungal endophytes were generally found to be closely related to plant pathogens, either as biotrophic or necrotrophic fungi, which was also applicable to *C. fructicola*; as with any other plant pathogenic *Colletotrichum* species, the fungus showed biotrophic and necrotrophic stages.

The switching to endophytic, biotrophic, and necrotrophic lifestyles in various fungal strains was analyzed by Delaye et al. [286] using a phylogenetic analysis of 5.8S rRNA gene sequences combined with ancestral character mapping. Based on the evolutionary scale, switching from an endophytic lifestyle to necrotrophy, and conversely, occurred multiple times at an equal frequency, whereas switching from an endophyte to a biotroph was infrequent. These findings imply that the disruption of mutualistic interactions resulted in a pathogenic lifestyle [287,288]. The phylogenetic analysis also showed that the endophytic stage is evolutionarily labile, with frequent switching occurring between endophytic and pathogenic states [275,289].

Clustering of the tested fungal species in the phylogenetic tree showed that five clusters contained only biotrophs, suggesting that once biotrophy had developed, reversal to an endophytic state or necrotrophy would not occur. The clustering of biotrophs also indicated that they were often depicted as having derived and evolutionarily stable traits. Endophytes and necrotrophs clustered together in most of the major clades, which indicated lifestyle switching at a very short evolutionary timescale [286]. Both endophytic and necrotrophic lifestyles have been reported for species within the gloeosporioides complex, such as *C. musae* and *C. gloeosporioides* [254,271,290].

Phylogenetic analysis based on multiple markers was performed on neotropical strains of endophytic and pathogenic *Colletotrichum* isolated from the leaves of cacao (*T. cacao*) and other plant species. Phylogenetic analysis revealed that the endophytic and pathogenic strains were clustered into two separate clades. The endophyte clade consisted of strains from cacao and other plants, whereas the pathogenic clades contained strains from a single host plant [19]. The two species, *C. tropicale* and *C. ignotum* (syn. *C. fructicola*), were the most common endophytes associated with cacao and other plants, whereas *C. theobromicola* was associated with anthracnose in cacao fruit and leaves. These findings suggest that endophytes do not show host-specificity, as they occur in various host plants, and that pathogens may have specialized relationships with host plants [19]. Host-specificity was also not detected among endophytic *Colletotrichum* isolated from the leaves of 12 tree species [291].

## 9. Bioactivity of *Colletotrichum fructicola*

Plant pathogenic and endophytic *Colletotrichum* spp. produce a variety of secondary metabolites with extensive biological activities. Secondary metabolites produced by plant pathogenic *Colletotrichum* play a role as pathogenicity and virulence factors during pathogenesis. Secondary metabolites produced by endophytic fungi may intensify host plant resistance against diseases, abiotic stress, insect infestation, and herbivory [292].

According to Moraga et al. [293], approximately 189 secondary metabolites have been recovered from *Colletotrichum* spp. and were biologically and chemically characterized. The most common secondary metabolites produced are sterols, terpenes, phenolics, pyrones, fatty acids, and nitrogen-containing compounds [294]. These secondary metabolites have potential applications in the agriculture and pharmaceutics industries as antimicrobial compounds, antiherbicides, and drugs to treat maladies.

To date, only two reports on the secondary metabolite compounds produced by *C. fructicola* have been made (Table 19). Endophytic *C. fructicola* recovered from *Nothapodytes nimmoniana* could synthesize camptothecin, an anticancer compound, under submerged conditions. The presence of camptothecin was detected using TLC and HPLC [295]. In another study, the endophytic *C. fructicola* isolated from the leaves of *Co. arabica* could produce indole-3-acetic acid (IAA) in vitro. A crude extract of the IAA was tested for plant growth ability in corn (*Zea mays* L.), rice (*Oryza sativa* L.), and rye (*Secale cereale* L.). Application of the IAA crude extract resulted in the elongation of the coleoptile segment of the tested plants, indicating that IAA can stimulate plant growth [296].

Based on the whole-genome sequence data of *C. fructicola* N425 causing anthracnose in tea plants and *C. fructicola* causing circular leaf spot in rubber trees, gene clusters related to secondary metabolites were detected. These secondary metabolite gene clusters may be involved in host-specific interactions and the pathogenicity of *C. fructicola* [232,298]. The findings demonstrated that *C. fructicola* has the capability to produce diverse groups of secondary metabolites.

In addition to the gene clusters related to secondary metabolites, numerous unique pectinases were detected in the genomes of dicot plants infected by *C. fructicola*. The genome of the pathogen encoded more than 100 pectinases [257]. Among the *Colletotrichum* spp. genome sequences, that of *C. fructicola* contained the largest number of cell wall-degrading enzymes [299]. The expression of a large number of extracellular enzymes might reflect the ability of *C. fructicola* to colonize a wide host range and adapt to various environments [257].

Cell wall-degrading enzymes, including pectinases, cutinases, hemicellulases, and cellulases, are involved in the penetration and colonization of host plants [300,301,302]. The production of extracellular enzymes, namely pectin lyase, polygalacturonase, and laccase, has been reported for *C. fructicola* causing apple bitter rot and Glomerella leaf spot. The production of different extracellular enzymes by the pathogen could also be related to the different symptoms observed in apple fruits and leaves [297].

Plant pathogens produce toxic metabolites that induce diseases and are involved in the development of disease symptoms [303]. Toxic metabolites can also act as virulence factors involved in pathogenicity [304,305]. Thus far, no reports on *C. fructicola*-produced toxic metabolites have been made. However, several phytotoxins have been detected from several *Colletotrichum* spp. that are involved in disease development. Colletotrichins A, B, and C were recovered from *C. nicotianae* cultures, and when applied to tobacco, lettuce, and rice seedlings, symptoms similar to those of anthracnose appeared [306,307]. From the culture filtrates of *C. higginsianum*, colletophyrandione was isolated, which was found to affect *Sonchus arvensis*, *Helianthus annuus*, *Convolvulus arvensis*, and *Ambrosia artemisiifolia* [308]. Chlororesorcinol recovered from *C. higginsianum* and produced necrosis on the leaves of *S. arvensis* and tomato plants. In addition, this compound showed potential herbicidal activity [308].

## 10. Management of Plant Diseases Caused by *Colletotrichum fructicola*

Chemical control is the main method used by many growers, which relies on the use of different classes of fungicides. However, the improper use of fungicides has resulted in toxic effects in humans and the generation of resistant species [309,310]. This has led to the development of more environmentally friendly methods, including biocontrol, the use of essential oils, and heat treatment.

### 10.1. Chemical Control

Chemical control using fungicides with different modes of action is frequently applied to control anthracnose in various crops. The fungicide classes commonly used to control *Colletotrichum* spp. associated with anthracnose was previously reviewed by Dowling et al. [311]. The most frequently used fungicide classes for controlling anthracnose are methyl benzimidazole carbamates (MBCs), dithiocarbamates, and quinone outside inhibitors (QoIs); however, due to increased fungal resistance, QoIs and MBCs are no longer effective [312,313,314].

For the proper management of anthracnose caused by *C. fructicola*, several screening studies of fungicide sensitivity and efficacy, as well as field trials, have been conducted on strawberries, pears, apples, peaches, and chili fruit (Table 20). The fungicide sensitivity study results provide information on the application and implementation of suitable fungicides for their effective usage and effective control of anthracnose. Fungicide sensitivity also provides information on the risk of resistance and status of the tested fungicides.

### 10.2. Essential Oils

Essential oils are used as an alternative method to control postharvest diseases, particularly for pathogen-infected fruit crops. Essential oils are commonly extracted from plants and contain various types of bioactive compounds. These bioactive compounds act synergistically to exhibit antioxidant, antimicrobial, and insecticidal properties [317,318,319]. The use of essential oils to control postharvest diseases is often combined with edible coatings, such as chitosan, which act as carriers of the bioactive compounds to increase their antimicrobial properties and contribute to a longer shelf life [320]. The use of essential oils to control postharvest diseases caused by *Colletotrichum* spp. in fruit crops was previously reviewed by da Costa Goncalves et al. [320].

Studies on the use of essential oils to control anthracnose and leaf spot caused by *C. fructicola* in several fruit crops are presented in Table 21. Several essential oils from lemongrass and mint, such as carvacrol, honokiol, magnolol, thymol, and magnolol, have the potential to inhibit *C. fructicola* growth and reduce the development of anthracnose lesions (Table 21). Although the use of essential oils is promising, further studies on their toxicity, safety levels, storage conditions, antifungal mechanisms, and cost-effectiveness are needed [300].

### 10.3. Biological and Other Control Methods

Many biocontrol agents, including avirulent *Colletotrichum*, bacteria, filamentous fungi, and yeast, have been tested against *Colletotrichum* spp. that cause anthracnose in postharvest fruit crops [322]. For the biocontrol of *C. fructicola*, the *Lysobacter enzymogenes* OH11 and *Bacillus tequilensis* YYC 155 strains showed the potential to control anthracnose in pears and the leaves of Ca. *oleifera*, respectively (Table 22).

Similar to essential oils, the use of biocontrol agents against *Colletotrichum* spp. still lacks information regarding the mechanism underlying antimicrobial inhibition, microbial toxicity and viability, storage conditions, and cost-effectiveness [322]. Other methods tested to control *C. fructicola* in fruit crops are neutral electrolyzed water, hot water and chitosan, and natamycin (Table 22). These methods have shown the potential to control anthracnose formation on strawberries, papaya, and apples [323,324,325].

**Table 22:** Biocontrol and other methods tested to control *Colletotrichum fructicola* infections in various crops.

	Diseases/Crops	Results	References
Biocontrol Methods			
*Lysobacter enzymogenes* OH11 - In vitro antimicrobial activity: 0.57 ± 0.13 cm (antagonistic activity) - Protective efficacy: reduced lesion diameter to 1.30 ± 0.49 cm (control: 2.0 ± 0.16 cm); >35% reduction and reduced severity - Most active extracellular enzyme producer; >protease, chitinase, cellulase, and glucanase activities	Pear anthracnose	Potential biocontrol agent against *C. fructicola* causing pear anthracnose and could help reduce the use of fungicides	[326]
*Bacillus tequilensis* strain YYC 155 - 45.35% mycelial growth inhibition - Reduced anthracnose lesion development in inoculated leaves - Strong ability to form a biofilm after 24–72 h; >colonization of leaf surface	*Camellia oleifera* leaf anthracnose	Potential biocontrol agent against *C. fructicola* causing leaf anthracnose of *Camellia oleifera*	[327]
Neutral electrolyzed water (pH 6.5–7.5): Applied through an overhead irrigation system	Strawberry anthracnose	Effective to control strawberry anthracnose	[323]
Hot water and chitosan Hot water dip at 49 °C for 20 min combined with 1% and 2% chitosan	Papaya anthracnose	Possibly used to control papaya anthracnose during postharvest storage without exerting negative effects on fruit physicochemical quality	[324]
Natamycin - Mycelia and spores were completely inhibited at 10 mg/L (MIC value). - Preventive and curative tests; >95% efficacies were obtained at 800 mg/L	Apple postharvest rot	Natamycin has the potential to be used as a biopreservative against *C. fructicola* infection in apples	[325]

Similar to the other infections caused by *Colletotrichum* species, control measures used to manage infections by *C. fructicola* can be specific depending on the host plant; however, some measures are generally applied to most host plants. To control anthracnose and leaf spot across a range of plants, an integrated approach is typically applied, which includes cultural practices, fungicide use, and biocontrol. Cultural practices have been employed to reduce inoculum production. The use of appropriate cultural practices and suitable fungicides could help improve disease management. Many biocontrol agents have been tested in vitro; however, their field efficacy has not been encouraging thus far, although they have been combined with chemical control methods in some trials.

In general, formulating effective control methods to manage anthracnose caused by *Colletotrichum* spp. requires relevant data and an understanding of the host plant, species of the causal pathogen, and the environment. According to Dowling et al. [311], these three factors are not easy to establish, and they had proposed three essential aspects that can enhance the understanding of plant pathogenic *Colletotrichum* spp. that could eventually lead to better disease management strategies. The first aspect involves identification of the causal pathogen, which is essential for establishing strategic disease management. As the genus *Colletotrichum* consists of several species’ complexes, distinguishing individual species is important due to differences in species-specific sensitivity profiles. The second aspect involves the extensive or large-scale field sampling of *Colletotrichum* isolates. A larger collection of isolates would provide precise data for developing suitable plant disease management strategies. The third aspect comprises fungicide sensitivity testing, which should include fungicides of different classes to provide a better formulation of disease management strategies. Fungicide sensitivity testing would also uncover potential threats of resistance or reduced sensitivity to a particular fungicide, as well as the inherent resistances of individual species Dowling et al. [311].

## 11. Conclusions and Future Directions

This review highlighted the widespread occurrence of *C. fructicola* in tropical, subtropical, and temperate regions, which is likely attributable to the increasing trade and movement of agricultural crops and plant materials. From current studies available, *C. fructicola* was demonstrated to adopt endophytic and pathogenic lifestyles that contribute to its adaptation and distribution. Many disease notes, new disease reports, and publications have reported new plant host infected by *C. fructicola* in several regions worldwide, indicating the expansion of its host and geographical areas. Similar to other plant pathogens, the host plant expansion of *C. fructicola* could be associated with the availability of a suitable host and climatic conditions that are favorable for its growth and establishment. Control measures used to manage *C. fructicola* infections still rely on fungicides; nevertheless, for effective disease management, an integrated approach is required to reduce disease incidence and severity.

The transition from endophytic to pathogenic behavior in *C. fructicola* presents an opportunity to exploit its endophytic form for biological control and disease suppression. In plants that are nonhosts or less susceptible to infection, the fungal endophyte survives in an endophytic manner and offer defense against various pathogens [256]. Additionally, the ability of symbiotic plasticity enables certain strains to switch between endophytic to pathogenic behavior depending on environmental factors and host plant growth stages [256].

Studies on closely related *Colletotrichum* species offer promising insights into the possibility of applying endophytic *C. fructicola* for disease suppression and biocontrol. For example, endophytic *C. gloeosporioides* showed antagonistic activity against fungal pathogens of *Camellia sinensis*, which may play a role in plant defense mechanisms against the pathogen [328]. In a study by da Silva Santos et al. [329], strains of endophytic *C. siamense* demonstrated growth promoting traits and suppressed the growth of *Fusarium oxysporum* in tomato plants. The main challenge in utilizing endophytic *C. fructicola* for disease suppression and biological control arise from its potential to act as a pathogen. The transition from being an endophyte to pathogen can frequently be initiated by changes in environmental conditions or the health status of the host plant. Thus, any use of endophytic *C. fructicola* as a biological control agent would necessitate a comprehensive understanding of the genetic and environmental factors that influence its lifestyle [284].

RNA interference (RNAi), a method for silencing genes, offers potential for the management of diseases caused by *C. fructicola*. The application of RNAi in managing *Colletotrichum* spp. infections has been reported by Gu et al. [330], Mahto et al. [331], Qiao et al. [332], and Goulin et al. [333]. The results from these studies are pertinent to *C. fructicola*, as RNAi can be utilized to target and silence essential genes that hinder the pathogen growth and ability to cause disease. Among the essential target genes for the pathogen’s development and pathogenicity are those involved in the formation of conidia and appressoria, cell wall biosynthesis, and pathogenicity factors [242,331]. Despite the promising application in disease management, several challenges must be resolved for RNAi to be widely adopted, including ensuring effective uptake by fungal cells and accurately targeting genes to prevent silencing of nontarget genes. Additionally, protocols for large-scale application and the necessary regulatory approvals are still being developed [334]. Future studies should focus on enhancing delivery mechanisms, such as through nanoparticle carriers, identifying specific target genes, and integrating RNAi with other strategies for integrated disease management [334].

Genomic comparisons of *C. fructicola* with other species of the gloeosporioides complex and other omics data, such as transcriptomics, provide a more comprehensive understanding of the ability of *C. fructicola* to infect a large number of hosts, the infection process, adaptation patterns, and insight into its transformation from endophytic to pathogenic lifestyles. To date, four genomes of *C. fructicola* have been sequenced, including *C. fructicola* Nara-gc5 causing strawberry crown rot [207], *C. fructicola* N425 that can infect tea plants [219], *C*. *fructicola* 1104–7 isolated from lesions of 320 leaf spot [232], and *C. fructicola* causing circular leaf spot in rubber trees [335]. In addition, three draft genomes of *C. fructicola* have been reported, namely of isolates associated with mango anthracnose [336], strawberry anthracnose [337], and tea leaf spot [298]. Thus far, no data exists on the genome sequence of endophytic *C. fructicola*. Nevertheless, the genomic and transcriptomic data of pathogenic *C. fructicola* provides notable information on the genes encoding secondary metabolites, effectors, and pectin-degrading enzymes that are associated with the transformation from the endophytic to pathogenic stages.

## Figures and Tables

**Table 1:** Common symptoms of anthracnose on fruits, leaves, and stems.

Plant Part	Symptom
Fruits 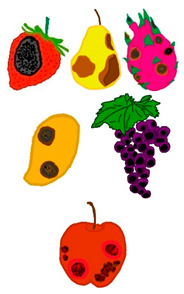	- Initial stage: Formation of small, water-soaked spots on the fruit surface. - Dark, sunken lesions: Often appeared as small, circular, or irregular spots that are initially light brown and slightly sunken. These lesions can enlarge over time, becoming a darker brown and water soaked. - Fruit rot: As the rot lesion progresses, it can lead to extensive rotting. - Acervuli with conidial masses: Under favorable conditions, such as high humidity, small, dark acervuli may form within the necrotic lesions. Masses of conidia are formed as yellowish to pinkish mucilaginous. - Premature fruit drop: Severe infections can cause fruits to drop prematurely. - Bitter rot: On apples and pears, the symptoms are characterized by light brown, slightly sunken lesions that enlarge and darken. References: [33,34]
Leaves 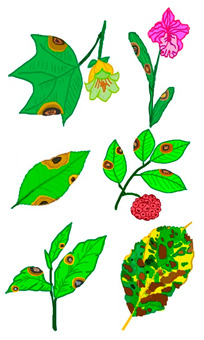	- Initial symptoms: Irregular dark brown to black leaf spots. These spots vary in size and shape, often appearing as irregular, necrotic areas. - These spots expand into larger, necrotic lesions. The center of the spots may become greyish to light brown, often surrounded by a darker, sometimes reddish-purple, border. - Necrotic lesions: As the disease progresses, the spots become more pronounced. Multiple spots merge to form larger, irregular necrotic areas, severely reducing the photosynthetic leaf area. - Acervuli: As the disease progresses, tiny black dots containing masses of yellowish to pinkish conidia may become visible within the lesions, especially under humid conditions. References: [33,34]
Stems/Twig 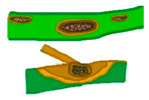	- Lesions: Symptoms appear as necrotic lesions, dark brown, and sunken. - Dieback: Infections can lead to twig dieback, especially of terminal leader shoots, and in severe cases, branch wilting [35]. - Cankers: In some hosts, the infection can result in sunken spots and cankers on stems, twigs, and branches [36].

**Table 2:** Diseases in apples caused by plant pathogenic *Colletotrichum fructicola*.

Diseases	Countries	References
Glomerella leaf spot	Brazil (culture collection of the Federal University of Santa Catarina) and Uruguay (culture collection of the University of the Republic)	[38]
Glomerella leaf spot and bitter rot	Uruguay	[39]
Glomerella leaf spot	Southern Brazil, Brazil, Uruguay	[34,37]
Glomerella leaf spot and bitter rot	Liaoning, Shandong, Henan, and Shaanxi, China	[47]
Bitter rot	Henan Province, China	[48]
Bitter rot (Honeycrisp, Jona Gold, Jonathan, Gibson Golden, Empire, Pink Lady, and Granny Smith)	Kentucky, USA	[49]
Bitter rot	Japan	[50]
Bitter rot	Republic of Korea	[51]
Bitter rot	Andong, Republic of Korea	[52]
Bitter rot (Joya Cripps Red, Granny Smith, and Pink Lady)	Region of Occitanie, France	[41]
Bitter rot	Cheongsong, Republic of Korea	[53]
Anthracnose (Fuji, Granny Smith, Red Delicious, Cripps Pink, and Early Red One)	Montevideo, Canelones, Uruguay	[43]
Anthracnose (Fuji)	Sangju, Republic of Korea	[44]
Anthracnose (Fuji, Tsugaru, and Hongro)	Gyeongbuk Province, Republic of Korea	[45]
Fruit rot (Pink Lady)	Emilia-Romagna (Northern Italy)	[46]

**Table 3:** Ripe rot and anthracnose in grapes caused by plant pathogenic *Colletotrichum fructicola*.

Species	Diseases	Countries	References
Grapes (*Vitis vinifera*)	Anthracnose/ripe rot (fruit and leaf)	Guizhou and Yunnan Provinces, China	[60]
Grapes (*V. vinifera*)	Anthracnose (leaf and shoot)	Santa Catarina State, Southern Brazil	[61]
Shine muscat (*V. labruscana* × *V. vinifera*)	Anthracnose (fruit)	Daegu, Republic of Korea	[62]
Grapes (*V. labrusca* and *V*. *vinifera*)	Ripe rot (fruit)	Southern Brazil	[59]
Grapes (*V. vinifera*)	Ripe rot (fruit)	Nagano Prefecture, Japan	[63]
Grapes (*V. vinifera* ‘Kyoho’)	Anthracnose (fruit)	Hangzhou, Taizhou, Jinhua, and Shaoxing (Zhejiang Province, China)	[57]

**Table 4:** Diseases in pears caused by plant pathogenic *Colletotrichum fructicola*.

Species	Diseases	Countries	References
Pear (*Pyrus pyrifolia* and *P. bretschneideri*)	Black spot/bitter rot (fruit)	Anhui, Fujian, Hubei, Jiangsu, Jiangxi, and Zhejiang, China	[64]
Pear (*P. bretschneideri* Rehd. var. ‘Suli’)	Black spot/bitter rot (fruit)	Dangshan County, Anhui Province, China	[65]
Pear (*P. pyrifolia* × *P. communis*)	Anthracnose (fruit)	Naju, Jeonnam Province, Republic of Korea	[66]
Pear (*P. bretschneideri*)	Bitter rot (fruit)	Dangshan County, Anhui Province, China	[67]
Sandy pear (*P. pyrifolia* Nakai)	Leaf black spot (leaf)	Southern China	[68]

**Table 5:** Anthracnose in *Citrus* spp. caused by plant pathogenic *Colletotrichum fructicola*.

*Citrus* spp.	Diseases	Countries	References
*Citrus bergamia* and *Ci. grandis*	Anthracnose (leaf)	Guizhou and Yunnan Provinces, China.	[69]
*Citrus* spp. and *Fortunella* spp.	Anthracnose (fruit, leaf, and shoot)	Zhejiang, Jiangxi, Guangdong, Guangxi, Yunnan, Fujian, and Shaanxi Provinces (China)	[70]
*Ci. sinensis*	Anthracnose (fruit)	Shimen, Changde, Hunan Province, China	[71]
*Citrus* spp.	Anthracnose (leaf, fruit, and stem)	Golestan, Mazandaran, Guilan, and Kerman Provinces, Iran	[73]
*Citrus* spp.	Anthracnose (fruit and leaf)	Tunisia	[74]
*Ci. reticulata*	Anthracnose (fruit)	Australia	[75]

**Table 6:** Anthracnose and crown rot of strawberry caused by plant pathogenic *Colletotrichum fructicola*.

Diseases	Countries	References
Anthracnose (fruit, leaf, and seedling)	Anhui, Hainan, Hebei, Hubei, Liaoning, and Shandong Provinces, and Beijing, Shanghai, China	[83]
Anthracnose (leaf and petiole)	Hubei, China	[84]
Anthracnose (diseased plant)	Chiba Prefecture, Japan	[79]
Anthracnose (leaf)	Shandong Province, China	[85]
Anthracnose (leaf and stolon)	Sichuan Province, China	[78]
Anthracnose (leaf)	Zhejiang Province, China	[86]
Anthracnose (leaf and petiole)	Eastern China	[77]
Anthracnose (leaf)	Miaoli, Hsinchu, Nantou, and Chiayi Counties, Taiwan	[87]
Anthracnose (leaf and crown)	Jiande and Zhoushan, Zhejiang Province, China	[88]
Crown rot	Zhoushan, Jiande, and Yuhang, Zhejiang Province, China	[81]
Crown rot	Florida, USA	[82]

**Table 7:** Anthracnose and other diseases of fruit crops caused by plant pathogenic *Colletotrichum fructicola*.

Fruit Crops	Diseases	Countries	References
Mango (*Mangifera indica*)	Anthracnose (fruit)	Aurangabad Maharashtra, India	[90]
	Anthracnose (fruit)	Oaxaca State, Mexico	[91]
	Anthracnose (fruit)	Brazil	[92]
	Anthracnose (fruit)	São Francisco Valley, Assú Valley, and Zona da Mata Pernambucana, northeastern Brazil	[93]
	Anthracnose (fruit)	Uttar Pradesh, Delhi, Chandigarh U.T., Gujarat, Maharashtra, and Goa States, India	[15]
	Anthracnose (leaf and fruit)	Guangxi, China	[94]
	Anthracnose (fruit)	Jeju, Republic of Korea	[95]
	Anthracnose (fruit)	Mexico	[96]
	Anthracnose (leaf)	Luzon, Visayas, and Mindanao, Philippines	[97]
	Black spot (young leaves)	Wady El-Mollak, Egypt	[98]
	Anthracnose (fruit)	Taiwan	[99]
Rambutan (*Nephelium lappaceum*)	Fruit rot	Puerto Rico	[100]
Pineapple (*Ananas comosus*)	Fruit rot and leaf-tip dieback	Northern Thailand	[101]
Watermelon (*Citrullus lanatus*)	Anthracnose (fruit, stem, leaf)	Henan, Jiangsu, Zhejiang, Jilin, Liaoning, Hebei, Jiangxi, and Hainan, China	[102]
Passion fruit (*Passiflora edulis*)	Anthracnose (fruit)	Zhenfeng, Qianxinan, and Guizhou Provinces, China	[103]
Dragon fruits (*Hylocereus undatus* and *H. monacanthus*)	Anthracnose	Philippines	[104]
Fig (*Ficus carica* L.)	Leaf blight	Malaysia	[105]
Cherry (*Prunus avium*)	Leaf spot/ anthracnose	Taizhou Academy of Agriculture Sciences, Zhejiang, China.	[106]
	Leaf spot	Beijing City, Sichuan, Shandong, and Liaoning Provinces, China	[107]
Persimmon (*Diospyros kaki*)	Anthracnose (young twig, fruit, and flower)	São Paulo and Paraná States, Brazil	[108]
	Anthracnose (fruit)	Philippines	[109]
Avocado (*Persea americana*)	Anthracnose (fruit)	Taiwan	[110]
	Anthracnose (leaf and fruit)	Chiang Rai Province, Thailand	[111]
Indian jujube (*Ziziphus mauritiana*)	Leaf spot	Nanning, Guangxi, China	[112]
Jute-leaved raspberry (*Rubus corchorifolius*)	Leaf spot	Longquan County, Zhejiang Province, China	[113]
Eggfruit/canistel (*Pouteria campechiana*)	Leaf spot	Baoshan, Yunnan, China	[114]
Chinese bayberry (*Myrica rubra*)	Leaf spot	Jiujiang City, Jiangxi Province, China	[115]
Carambola (*Averrhoa carambola*)	Anthracnose (fruit)	Yuancun District, Guangzhou, China	[116]
Pomegranate (*Punica granatum* L.)	Leaf spot/ anthracnose	Jiangxi Agricultural University, Nanchang, Jiangxi Province, China	[117]
Litchi (*Litchi chinensis* Sonn.)	Anthracnose (leaf)	Qinzhou City, Guangxi Province, Southern China	[118]
Kiwifruit (*Actinidia deliciosa*)	Anthracnose (leaf and fruit)	Kagawa Prefecture, Japan	[119]
Siberian apricot (*Prunus sibirica* L.)	Shot hole leaf	Chengdu, Sichuan Province, China	[89]

**Table 8:** Anthracnose and fruit rot of coffee berries caused by plant pathogenic *Colletotrichum fructicola*.

Diseases	Countries	References
Anthracnose (berries)	Northern Thailand	[18]
Fruit rot (berries)	Puerto Rico	[120]

**Table 9:** Anthracnose of *Camellia* spp. caused by plant pathogenic *Colletotrichum fructicola*.

*Camellia* spp.	Diseases	Countries	References
*Camellia sinensis*	Anthracnose (leaf)	Fujian Province, China	[125]
	Anthracnose (leaf)	China	[6]
	Anthracnose (leaf)	China	[126]
	Anthracnose (leaf)	Zhejiang Province, China.	[127]
	Anthracnose (leaf)	Guanxi Township, Hsinchu County, Taiwan	[123]
	Brown blight (foliar blight)	Taiwan	[128]
*Ca*. *yuhsienensis*	Anthracnose (leaf)	Youxian, Zhuzhou, Hunan Province, China	[124]
*Ca*. *oleifera*	Anthracnose (leaf and fruit)	Southern China	[129]
	Anthracnose (leaf)	Wenchang, Qiongzhong, and Wuzhishan, Hainan Province, China	[130]
*Camellia* spp.	Anthracnose (leaf)	Jiaoling County, Guangdong Province, China	[122]
*Ca. chrysantha*	Anthracnose (leaf)	Fangchenggang City, Guangxi Zhuang Autonomous Region of China	[131]
*Ca*. *oleifera*, *Ca*. *sinensis*, and *Ca*. *japonica*	Anthracnose (leaf)	Zhejiang, Jiangxi, Yunnan, and Shanghai, China	[132]

**Table 10:** Anthracnose and fruit rot of vegetable crops caused by plant pathogenic *Colletotrichum fructicola*.

Vegetable Crops	Diseases	Countries	References
Chili (*Capsicum annuum*)	Anthracnose (fruit)	Southern India	[133]
*Cap. annuum* var. Arka Lohit	Anthracnose	Karnataka, India	[134]
*Capsicum* spp.	Anthracnose	Sichuan Province, China	[135]
*Capsicum* spp.	Anthracnose (fruit and leaf)	29 districts in China	[136]
*Cap. annuum*	Fruit rot	Northwestern Himalayan Region, India	[137]
Green and red *Cap. annuum* and *Cap. frutescens*	Anthracnose	Peninsula Malaysia	[138]
*Cap. annuum*	Anthracnose	Fujian, China	[139]
*Capsicum* spp.	Anthracnose (fruit)	North-Central Vietnam	[140]
Culinary melon (*Cucumis melo* var. *acidulus*)	Anthracnose (leaf spot)	Thiruvananthapuram District, Kerala, India	[141]
Japanese pickling melon (*Cucumis melo* var. *conomon*)	Anthracnose (fruit)	Kyoto, Japan	[142]
Luffa sponge gourd (*Luffa cylindrica*)	Anthracnose (leaf)	Hunan Province, China	[143]
Chinese flowering cabbage *(Brassica parachinensis*)	Anthracnose (leaf)	Guangdong Province, southern China	[144]

**Table 11:** Ornamental plants infected by plant pathogenic *Colletotrichum fructicola*.

Ornamentals	Diseases	Countries	References
*Orchidaceae*			
*Cattleya*	Anthracnose (leaf)	Alagoas, northeastern region of Brazil	[145]
*Phalaenopsis*	Anthracnose (petal)	Alagoas, northeastern region of Brazil	[145]
*Bletilla striata*	Anthracnose (leaf)	Zunyi and Xingyi, Guizhou Province, China	[146]
*Bletilla striata*	Leaf spot/Anthracnose	Guilin, Guangxi Province, China	[147]
*Dendrobium officinale*	Anthracnose (leaf)	Ningguo City, Anhui Province, China	[148]
*Crinum asiaticum*	Leaf and stem rot	Nanning Botanical Garden of Medicinal Plants, Guangxi Province, China	[149]
Other ornamental plants			
*Salvia leucantha* (Mexican bush sage), *S. nemorosa* (woodland sage), and *S. greggii* (Autumn sage)	Leaf spot/ anthracnose	Northern Italy	[150]
*Mandevilla* × *amabilis*	Anthracnose (leaf)	Nanning, Guangxi, China	[151]
*Ceanothus thyrsiflorus* (blue blossom), *Hydrangea paniculata* (hortensia), *Cyclamen persicum* (cyclamen), and *Liquidambar* *styraciflua* (American sweetgum)	Leaf spot/ anthracnose	Northern Italy	[152]
*Liriodendron chinense* × *tulipifera* (Chinese tulip tree)	Leaf spot	Nanjing Forestry University, Jiangsu Province, China	[153]
*Magnolia wufengensis* (China red)	Leaf spot	Yuyangguan Township, Wufeng County, Hubei Province, China	[154]
*Ixora chinensis* (Chinese Ixora)	Leaf spot/ anthracnose	Nanning, Guangxi, China	[155]
*Rosa chinensis* (China rose)	Leaf spot/ anthracnose	Nanyang Academy of Agricultural Sciences in Nanyang, Henan Province, China	[156]
*Paeonia lactiflora* (peony)	Anthracnose (leaf)	Poyang County, Shangrao City, Jiangxi Province, China	[157]
*Osmanthus fragrans* (fragrant olive)	Anthracnose (leaf)	Quanjiao, China	[158]
*Salix babylonica* (weeping willow)	Anthracnose (leaf)	Jiangsu, Shandong, Hubei Province, China	[159]

**Table 12:** Peanuts and tree nuts infected by plant pathogenic *Colletotrichum fructicola*.

Nuts	Diseases	Countries	References
Tree nuts			
Walnuts (*Juglans regia* L.)	Anthracnose (fruit and leaf)	Jinan, Shandong, China	[160]
Pecan (*Carya illinoinensis*)	Anthracnose (fruit and leaf)	Jiande, Zhejiang Province; Ji’an, Jiangxi Province; and Yuxi, Yunnan Province, China	[161]
Peanut			
Peanut (*Arachis hypogaea* L.)	Leaf spot/ anthracnose	Xuzhou Academy of Agriculture Sciences, Jiangsu, China	[162]
Macadamia (*Macadamia ternifolia*)	Anthracnose (leaf)	Changping, Fangshan, Haidian, Huairou, Mentougou, Miyun, and Pinggu: districts in Beijing, China	[163]
Chinese hickory (*Carya cathayensis*)	Leaf spot/ anthracnose	Huzhou, Zhejiang, China	[164]

**Table 13:** Medicinal plants infected by plant pathogenic *Colletotrichum fructicola*.

Medicinal Plants	Diseases	Countries	References
*Paris polyphylla* Smith var. *chinensis*	Leaf and stem rot	Guangze and Shaxian Counties, China	[165]
*Amomum villosum*	Leaf spot	Guangxi Province, China	[166]
*Kadsura coccinea*	Leaf spot	Longan, Guangxi, China	[167]
*Ficus hirta* (hairy fig)	Leaf spot/ anthracnose	Qinzhou and Zhanjiang Cities, China	[168]
*Smilax glabra* (sarsaparilla)	Leaf spot	Qinzhou City, Guangxi Province, China	[169]
*Callerya speciosa* (climbing shrub)	Leaf spot	Nanning, Guangxi, China	[170]
*Epimedium sagittatum*	Leaf spot	Zhumadian City, China	[171]

**Table 14:** Weeds infected by plant pathogenic *Colletotrichum fructicola*.

Weeds	Diseases	Countries	References
*Eichhornia crassipes* (water hyacinth)	Leaf spot, stem rot, crown rot, and petiole rot	Minjiang and Xiyuanjiang, Fuzhou, China	[172]
*Amaranthus blitum* (pigweed)	Leaf spot	Nara, Japan	[173]
*Bromus japonicus* Thunb. (Japanese brome)	Leaf spot/ anthracnose	Wuqing District, Tianjin, China	[174]

**Table 15:** Other plants or crops infected by *Colletotrichum fructicola*.

Crops/Plants	Diseases	Countries	References
Common bean (*Phaseolus vulgaris*) and cowpea (*Vigna unguiculata*)	Anthracnose	Mazandaran, Guilan, and Zanjan Provinces, Iran	[175]
Tobacco (*Nicotiana tabacum*)	Anthracnose (leaf)	Guizhou, China	[176]
White jute (*Corchorus capsularis*)	Anthracnose (leaf and stem)	Fujian, Henan, Guangxi, and Zhejiang Provinces, China	[177]
Japanese fatsia (*Fatsia japonica*)	Leaf spot	Fujian Province, China	[178]
Tree-like cactus (*Nopalea cochenillifera*)	Cladode brown spot	Pernambuco, Brazil	[179]
Climbing shrub (*Callerya speciosa*)	Leaf spot	Nanning, Guangxi, China	[170]
*Aesculus chinensis* (landscaping tree)	Leaf blotch	China	[180]
*Eucalyptus dunnii*, *Eu. nitens*, and *Eu. macarthurii*	Leaf spot	South Africa	[181]
*Dalbergia hupeana* (wood and medicinal tree)	Leaf spot	Jiangxi Province, China	[182]
Star anise (*Illicium verum*)	Leaf spot	Shanglin and Jinxiu Counties, Guangxi Province, China	[183]
Spotted laurel (*Aucuba japonica*)	Anthracnose (leaf)	Jeju Island, Republic of Korea	[184]
Rubber (*Hevea brasiliensis*)	Anthracnose (leaf)	Yunnan Province, China	[185]
Soybean (*Glycine max*)	Anthracnose (pod)	Chongzhou, Sichuan Province, China	[186]
Soybean	Anthracnose (stem)	Campos Novos, Santa Catarina, Brazil	[187]
Mangrove tree (*Rhizophora apiculata*)	Leaf spot	Thailand	[188]
Tree peony (*Paeonia delavayi*)	Leaf spot/ anthracnose	Yuxi, Yunnan Province, China	[189]
Oak (*Quercus acutissima*, *Q*. *mongolica*, and *Q*. *variabilis*)	Anthracnose (leaf)	Anhui, Hainan, Henan, Shaanxi, and Shandong Provinces, Inner Mongolia Autonomous Region, and Beijing City, China	[190]
*Manglietia decidua* (*Magnoliaceae*—deciduous tree)	Leaf spot/ anthracnose	Jiangxi Province, China	[191]
Sorghum (*Sorghum bicolor*)	Leaf spot	Guizhou Province, Southwest China	[192]
Torch ginger	Bract	Brazil	[193]

**Table 16:** Genes/proteins identified and characterized during *Colletotrichum fructicola* pathogenesis and their functions.

Genes/Proteins	Diseases/Host Plants	Functions	References
CfPMK1 [pathogenicity MAPK (PMK)]	Glomerella leaf spot (apple leaf)	Appressorium development, pathogenesis, sexual development, and stress tolerance	[205]
CfSnf1 (protein kinase)	Anthracnose (tea oil leaf)	Utilization of specific carbon sources, conidiation, appressorium formation, and stress responses	[208]
CfEC92 (effector)	Glomerella leaf spot (apple fruit and leaf)	Functional at an early stage of infection, appressorium-mediated penetration, differentiation of primary hyphae, and suppresses host plant defense reactions	[194]
CfSte12 (transcription factor)	Glomerella leaf spot (apple fruit and leaf)	Conidial germination, appressorium formation, appressorium-mediated penetration, colonization, and development of sexual reproductive structures	[209]
CfVAM7 (SNARE protein)	Anthracnose (tea oil leaf)	Hyphal growth, sporogenesis, appressorium formation, responses to stress, vacuole fusion, and pathogenicity	[210]
CfSet1 (H3K4 methyltransferase)	Anthracnose (tea oil leaf)	Vegetative growth, asexual reproduction, appressorium formation, and penetration	[211]
CfGcn5 (histone acetyltransferase)	Anthracnose (tea oil leaf)	Involved in ribosomes, catalytic and metabolic processes, primary metabolism, and autophagy	[212]
CfMcm1 (transcription factor)	Glomerella leaf spot (apple leaf)	Plays a role in pathogenicity, sexual and asexual reproduction, and melanin synthesis	[213]
CfAtg5 (autophagy-related protein)	Tea oil leaf and apple fruit	Required for autophagy, growth and conidiation, and appressoria formation	[214]
CfCpmd1 (transcription factor)	Glomerella leaf spot (apple leaf)	Strain compatibility during sexual reproduction, hyphal growth, sporulation, and formation of appressoria	[215]

**Table 17:** Cross-infection of *Colletotrichum* spp. from primary host to other plants.

*Colletotrichum* Species	Primary Host	Cross-Host Infection	References
*C. gloeosporioides*, *C. siamense*, *C. fructicola*, *C. truncatum*, *C. scovillei*, *C. brevisporum*, *C. sichuanensis*	chili	pear	[135]
*C. gloeosporioides*	avocado	mango	[235]
	mango	avocado	
*C asianum*	coffee	chili and rose apple	[236]
	mango	chili and mango	
*C. cordylinicola*	rose apples	guava, mango, chili, rose apple, papaya	
	*Cordyline fruticosa*	papaya	
*C. fructicola*	coffee	orange, chili, rose apple, papaya	
*C. fructicola*	papaya	orange, chili, rose apple	
*C. fructicola*	longan	mango, chili, rose apple, papaya	
*C. siamense*	coffee	orange, guava, mango, chili, papaya	
*C. siamense*	chili	guava, mango	
*C. simmondsii*	papaya	guava, mango, chili, rose apple	
*C. gloeosporioides*	acid lime, custard apple, pomegranate, cashew and guava	mango leaves and fruits	[238]
*C. gloeosporioides*	limonium	peach, pear, mango, nectarine, and strawberry	[239]
*C. acutatum*	strawberry	peach, pear, mango, nectarine, and strawberry	
*C. nymphaeae*, *C. siamense* *C. fioriniae*	strawberry	apple, blueberry	[240]
*C. fioriniae*	blueberry	apple, strawberry	
*C. fioriniae*	apple	blueberry, strawberry	
*C. gloeosporioides*	mango	tomato	[241]
*C. gloeosporioides*	avocado, mango	strawberry, pepper, guava, papaya	[242]

**Table 19:** Biocompounds and bioactivity of *Colletotrichum fructicola*.

Endophytic *C. fructicola*-Produced Biocompounds		
Host Plants	Biocompounds	References
*Nothapodytes nimmoniana*	Camptothecin	[295]
*Coffea arabica*	Indole-3-acetic acid	[296]
Bioactivity of pathogenic *C. fructicola*		
	Bioactivity	
Apple bitter rot and Glomerella leaf spot	Extracellular enzymes (pectin lyase, polygalacturonase, and laccase)	[297]

**Table 20:** Fungicide sensitivity testing to control *Colletotrichum fructicola* infections in various crops.

Control Methods	Diseases/Crops	Results	References
Fungicide sensitivity			
Mycelia growth - Difenoconazole (EC_50_): 0.08, 0.19, 0.11, and 0.20 µg/mL (four isolates)	Strawberry anthracnose (leaf)	Difenoconazole was the most effective for inhibiting the growth of *C. fructicola* isolates	[85]
Mycelia growth (µg/mL) - Difenoconazole: 0.036 ± 0.031 - Tebuconazole: 6.305 ± 0.903 - Prochloraz: 0.140 ± 0.074 l - Azoxystrobin: >100	Strawberry anthracnose (leaf, stolon, and crown)	Difenoconazole and tebuconazole could be used as alternative fungicides to prevent infections	[77]
Mycelia growth - Difenoconazole: 10% WG (EC_50_, 0.5579 mg/L) - Trifloxystrobin + tebuconazole 75% WG (EC_50_, 1.0354 mg/L)	Passion fruit anthracnose	Difenoconazole and trifloxystrobin + tebuconazole have the potential to prevent infections	[103]
Mycelia growth - Pyraclostrobin (EC_50_, 0.1722–0.9783 μg/mL) - Pyraclostrobin + salicylhydroxamic acid (EC_50_, 0.0978–0.7785 μg/mL) - Difenoconazole (EC_50_, 0.1792–2.6672 μg/mL)	Chilli fruit anthracnose	Inhibition of mycelial growth indicated pathogen sensitivity to the fungicides tested	[139]
Greenhouse trials (preventive efficacy) - Pyraclostrobin (73.02%) - Difenoconazole (75.71%) - Difenoconazole + pyraclostrobin (79.38%)		Fungicides tested showed good preventive efficacy to reduce anthracnose incidence	
Mycelia growth - Prochloraz (EC_50_, 0.02 μg ai/mL) - Pyraclostrobin (EC_50_, 0.31 μg ai/mL) - Tebuconazole (EC_50_, 0.55 μg ai/mL) Conidia germination - Prochloraz (EC_50_, 2.01 μg ai/mL) - Pyraclostrobin (EC_50_, 2.22 μg ai/mL) - Tebuconazole (EC_50_, 3.02 μg ai/mL)	Apple Glomerella leaf spot	Fungicides tested highly inhibited mycelial growth and conidia germination	[315]
Field trial Alternate application of pyraclostrobin and Bordeaux mixture		Alternate application of pyraclostrobin and Bordeaux mixture was highly effective in controlling the disease	
Mycelia growth - Prochloraz: no resistant isolates - Pyraclostrobin: highly resistant isolates, 74%; weakly resistant isolates, 26% - Procymidone and fludioxonil: 100% resistance (all isolates)	Peach anthracnose	Prochloraz has the potential to control peach anthracnose	[316]
Mycelia growth - Trifloxystrobin (30–55%) - Kresoxim-methyl (29–55%) - Tebuconazole (84–100%) - Metconazole (79–100%) - Fluazinam (87–100%) Conidia germination - Trifloxystrobin (62–100%) - Kresoxim-methyl (51–96%) - Tebuconazole (64–100%) - Metconazole (70–80%) - Fluazinam (94–100%)	Apple bitter rot	Provided information on fungicide sensitivity for the effective management of apple bitter rot	[53]

**Table 21:** Essential oil testing against *Colletotrichum fructicola* infections in various crops.

Essential Oils	Diseases/Crops	Results	References
Carvacrol: - Inhibited mycelial growth at 400 μL/L - Inhibited spore germination and germ tube elongation at 120 μL/L - Reduced the emergence and development of anthracnose in vivo	Red pitaya fruit anthracnose	Carvacrol showed antifungal activity and the potential to control anthracnose of the red pitaya fruit	[321]
Lemongrass (*Cymbopogon citratus*) combined with a chitosan coating inhibited fungal growth, as well as partial and total inhibition of lesion development	Postharvest guava, mango, and papaya	Possibly effective to control postharvest anthracnose development in tested fruits	[318]
Mint (*Mentha piperita*) combined with a chitosan coating inhibited mycelial growth and reduced the severity of anthracnose lesions during storage	Mango (Tommy Atkins) anthracnose	Alternative method for controlling anthracnose development during postharvest	[317]
Mycelial growth (sensitivity to fungicides): - Honokiol: EC_50_, 21.70 ± 0.81 µg/mL - Magnolol: EC_50_, 24.19 ± 0.49 µg/mL - Thymol: EC_50_, 31.97 ± 0.51 µg/mL - Carvacrol: EC_50_, 31.04 ± 0.891 µg/mL	Sorghum leaf spot	Honokiol, magnolol, thymol, and carvacrol inhibited mycelial growth, indicating good antifungal effects, with honokiol showing the most significant effect	[192]
Field trial: - Honokiol: severity (%), 6.61 (2021) and 5.66 (2022); yield (kg/16 m^2^), 13.33 ± 0.16 (2021) and 14.05 ± 0.41 (2022) - Magnolol: severity (%), 6.04 (2021) and 6.01 (2022); yield (kg/16 m^2^), 13.41 ± 0.21 (2021) and 14.19 ± 0.11 (2022)	Sorghum leaf spot	Honokiol and magnolol effectively controlled the disease and increased yield	[192]

## Data Availability

No new data were created or analyzed in this study. Data sharing is not applicable to this article.
